# Comparison of clinical and patient-reported outcomes of three procedures for recurrent anterior shoulder instability: arthroscopic Bankart repair, capsular shift, and open Latarjet

**DOI:** 10.1186/s13018-019-1340-5

**Published:** 2019-10-18

**Authors:** Yingjie Xu, Kailun Wu, Qianli Ma, Lei Zhang, Yong Zhang, Wu Xu, Jiong Jiong Guo

**Affiliations:** 1 Department of Orthopedics, The First Affiliated Hospital of Soochow University, Suzhou, 215006 China; 2 Department of Orthopedics, Suzhou Dushuhu Public Hospital (Dushuhu Public Hospital Affiliated to Soochow University), Suzhou, China; 3 Department of Orthopedics, Fuzhou Second Hospital Affiliated to Xiamen University, Fuzhou, China

**Keywords:** Shoulder, Instability, Bankart, Latarjet, Capsular shift

## Abstract

**Background:**

Best surgical of recurrent anterior shoulder instability remained controversial. We knew little about the superiority and choice between traditional open and modern arthroscopic techniques. We hypothesized that outcomes of all patients will be similar regardless of surgical technique.

**Methods:**

A retrospective case-cohort analysis of 168 patients who had recurrent anterior shoulder instability was conducted from September 2010 to December 2013. All cases (mean age 30.8 [range 18–50] years) were performed with arthroscopic Bankart repair (33 males/20 females), open Latarjet (34 males/18 females), and capsular shift (31 males/14 females). The average follow-up was 67.6 months (range 60–72). The shoulder instability index score (ISIS) was more than 3 with an average of 6.4.

**Results:**

All treatments proved to be effective in improving shoulder functional status and reducing symptoms, while Latarjet had an advantage over subjective perception. The Rowe scores in arthroscopic Bankart, open Latarjet, and capsular shift group were 92.3 ± 1.5, 96.2 ± 2.1, and 93.2 ± 2.3, respectively, with significant difference. There was no significant difference in other functional outcomes. However, the Latarjet group in subjective results (subjective shoulder value (SSV) and subjective shoulder value for sport practice (SSV Sport)) was superior to the others (*P* < 0.05). There were two relapsed cases in arthroscopic Bankart and capsular shift group, respectively, and no recurrence in open Latarjet group.

**Conclusion:**

Arthroscopic Bankart repair has the advantage of mini-invasion and rapid recovery. Capsular shift offers stabilizing of inferior or multidirectional type, especially for little bone defect. Latarjet was more effective in reducing recurrence with higher stability.

**Level of evidence:**

Therapeutic level III

## Introduction

Recurrent anterior shoulder instability may be caused by avulsion of the anterior glenoid rim and irreversible stretching of anteroinferior capsule, which is known as a Bankart lesion [1]. It has been reported that the incidence is up to 60% with increasing trend [2]. The optimal surgical treatment of recurrent anterior shoulder instability associated with severe glenoid defects and capsular deficiency remains challenging. It was a disabling condition commonly treated with arthroscopic Bankart repair, open Latarjet, or capsular shift [3].

With the development of arthroscopy, Bankart repair has been currently the preferred choice to treat recurrent instability with nearly 90% of surgeons accepted [4]. Several studies have shown that Bankart repair ensured greater stability and less recurrence compared with the others [5, 6]. However, opponents held that there were unmeasured confounding factors, which were probably related to differences in outcome, including sex, age, hyperlaxity, history of instability, level of sports, and lesions of glenoid and humeral bone. In addition, the recurrence of these techniques varied obviously in the literature [7]. They believed that surgical procedure should focus on more anatomic repairs, which Bankart repair may not. Today, we all knew that glenohumeral bone loss and capsular deficiency were considered to remain ubiquitous in anterior shoulder instability. It was known as Broca-Perthes-Bankart (a combination of capsular laxity and glenohumeral ligament avulsion) [5]. The Latarjet procedure has been shown to be a reliable technique which fixed the failings of Bankart repair and had a lower rate of recurrent instability [7]. Furthermore, the capsular shift was widely used in the USA because it rectified both the Bankart lesion and capsular laxity. These three procedures have become mainstream for recurrent anterior shoulder instability. Nevertheless, we did not have enough knowledge about the superiority between traditional open and modern arthroscopic techniques according to available findings. There was still no rational proposal so that the choice of treatments often depended on training or preferences.

It was very important for surgeons to acquire associated knowledge about Bankart and open surgery, which can provide accurate preoperative references of the risks and benefits. In this study, we evaluated the three treatments with a larger sample size and longer follow-up (at least 36 months). The aim of this study was to assess the functional and subjective results of these surgical and determine which one better suited our needs.

## Materials and methods

### Patients

Between September 2013 and December 2015, we performed a retrospective study to analyze the outcomes of recurrent anterior shoulder instability at our hospital. This study plan was approved by the local ethics committee, and the consent was obtained.

All patients had a diagnosis of recurrent anterior shoulder instability as the main symptom, who had dislocation of the shoulder joint with slight external force, for at least 1 year. In the meantime, we screened a random sample of subjects of 18–50 age group. Other inclusion criteria were subjects with a score of at least 3 on the instability severity index score (ISIS) [8], higher sports requirement (especially over-shoulder movement). Exclusion criteria included a condition other than osteoarthritis of the shoulder (significant changes in joint space), multiple recurrent shoulder subluxations or dislocations, first dislocated, severe epilepsy, unclosed osteoepiphysis, severe glenoid bone loss (glenoid loss of contour on anteroposterior radiograph), an active infection, and a major medical illness. Eventually, 102 patients met the above-defined criteria. Until now, 11 patients had incomplete data, leaving a cohort of 91 patients (mean age 30.8 [range 18–50] years, 62 right shoulders, mean ISIS 6.4) available for review at a minimum of 36 months. From these patients, three groups have been selected according to different surgical procedures (Bankart repair, Latarjet procedure, and capsular shift). Demographic data and preoperative characteristics had no statistical difference among the three groups (Table 1).

**Table 1:** Demographic data and preoperative characteristics

Variable	Bankart (*n* = 53)	Latarjet (*n* = 52)	Capsular shift (*n* = 45)
Age (years, mean ± SD)	29.81 ± 4.31	31.23 ± 6.12	30.75 ± 3.85
Male/female	33/20	34/18	31/14
Dominant involvement	37	32	31
Course of preoperative dislocation (months, mean ± SD)	14.25 ± 5.10	13.80 ± 3.13	13.42 ± 3.72
Competitive sport before instability (%)	26 (49.1%)	20 (38.5%)	24 (53.3%)
Hyperlaxity (ER > 85°) (%)	27 (51.0%)	24 (46.2%)	21 (46.7%)
Rowe score	48.74 ± 12.08	42.23 ± 14.20	50.87 ± 9.61
ISIS (mean ± SD)	6.16 ± 2.81	7.01 ± 3.02	6.50 ± 2.56

*ER* external rotation, *ISIS* instability severity index score. Competitive sport: ball games, throwing events, gymnastics, and so on

*P* < 0.05 was considered statisically signifcant

Latarjet procedure should be considered as the first choice if the loss of the anterior glenoid rim was larger than 50% of the maximum anteroposterior diameter of glenoid on routine computed tomography (CT) or magnetic resonance imaging (MRI) before surgery [7]. In other cases, a judgment decision of the operation was made by a surgeon.

### Operative technique

All operations were carried out by experienced shoulder surgeons. There were 35 patients in the Bankart repair group (22 males). The patient was placed in the lateral decubitus position, and the arm was abducted approximately 45° with longitudinal traction. When performing Bankart repair, the cartilage of the anterior border of the scapula was first cleaned and the anterior and posterior capsule sacral lip complex was freshened. Then, the damaged anterior inferior joint capsule sacral lip complex was completely released. The suture anchor (Lupine BR Anchor, DePuy Mitek, Raynham, MA, USA) was evenly placed on the cartilage surface of the glenoid for repair, and the suture was introduced into the labrum by a suturing device and knotted.

**Fig. 1:**
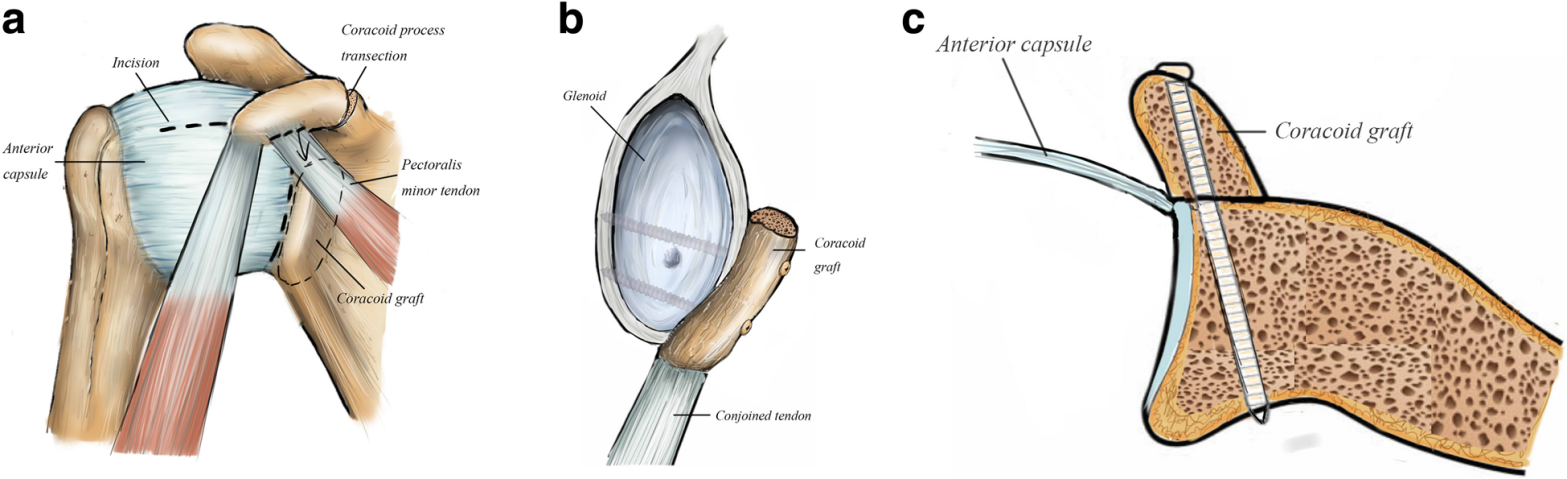
**a** An inverted L-shaped opening (dotted line) is made in the anterior approach to form the capsule flap from the glenoid neck. Pectoralis minor (dotted line) is detached from the coracoid before the coracoid osteotomy is carried out. **b** Coracoid graft is fixed to glenoid rim with 2 malleolar screws. If the curve is not fit, the graft can be re-sharpened. **c** Put the graft onto glenoid rim as an extension of the articular platform

There were 26 patients in the Latarjet group (17 males). The shoulder joint was in the abduction and external rotation, so that the coracoacromial ligament was revealed. The coracoacromial ligament was divided 1 cm lateral to the coracoid, and the coracoid was osteotomized at its base. Then, two drill holes were pre-drilled through the coracoid. The anteroinferior aspect of the labrum was excised. The coracoid bone was fixed to the glenoid rim through the subscapularis tendon with malleolar screws. The stump of the coracoacromial ligament was sutured to the most medial aspect of the joint capsule (Fig. 1).

**Fig. 2:**
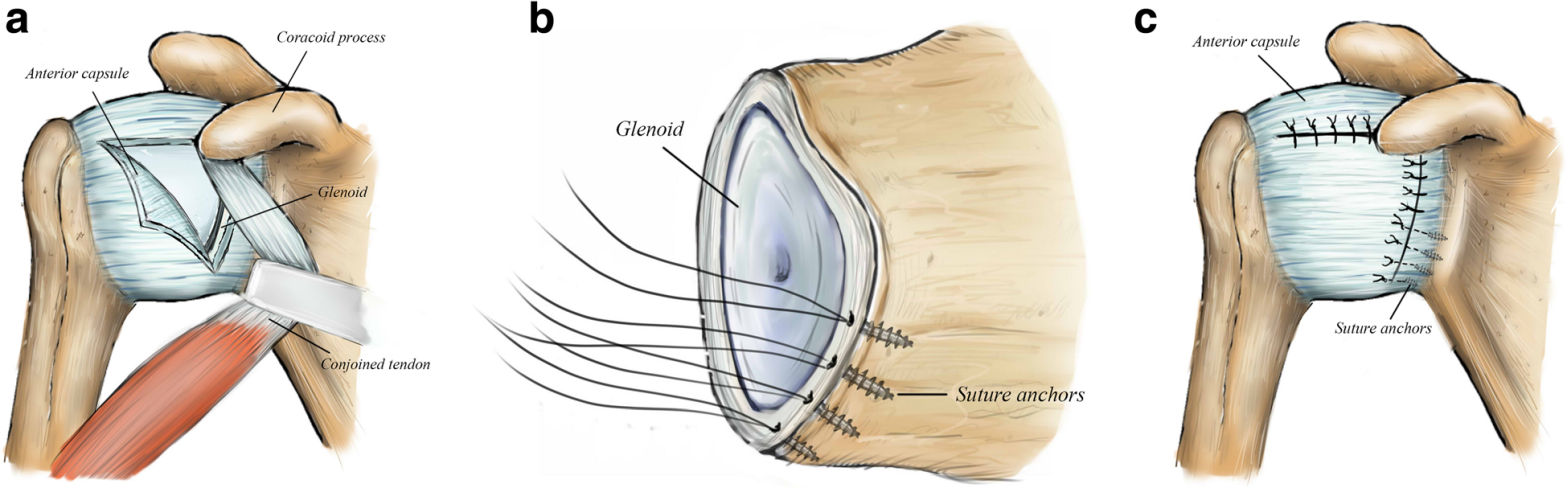
**a** The capsule is incised based on inverted L shape to expose glenoid rim adequately. **b** Three to four suture anchors are positioned medially from 3 o’clock to 6 o’clock to the direction. **c** Pull the flap superiorly to make incised capsule tied down to the glenoid edge

There were 30 patients in the capsular shift group (21 males). For capsular shift, the incision started several centimeters below the tip of the condyle and proceeded to the inferior border of the pectoralis major muscle. The subscapularis tendon was incised 1 cm medial to its insertion on the lesser tuberosity, and the muscular position of the subscapularis was also separated from the capsule. The capsular flaps were repaired. When the capsule had been sufficiently mobilized, the capsule was anchored medially to the glenoid with suture anchors (Mitek G2 Anchor, Raynham, MA, USA). The capsule was split in “T” or “L” fashion, then the inferior flap was pulled superiorly, and was sutured to the lateral capsular remnant. The capsular cleft between the superior and middle glenohumeral ligaments was closed and reinforced the capsule anteriorly (Fig. 2).

After surgery, the postoperative regime was the same for the three groups. Patients were immobilized in a shoulder brace in internal rotation for 6 weeks and applied ice around the shoulder joint within 3 days after surgery. Active motion exercises were carried out as tolerated to improve the range of motion and muscular strength by a physical therapist after 6 weeks.

### Outcome measures

All patients were assessed by one observer, independent of the operating surgeons, with a questionnaire that included stability, satisfaction, subjective shoulder value (SSV) [9], subjective shoulder value for sport practice (SSV Sport), the American Shoulder and Elbow Joint Surgery Association shoulder joint score (ASES), Rowe score, University of California at Los Angeles scoring system (UCLA score), and external rotation (ER). ER was measured with the elbow at 90° of abduction using a goniometer. Hyperlaxity was defined as the small resistance and large rotation angle of ER (ER > 85°). We ranked SSV and SSV Sport between 0 and 100% to assess activities of daily living [10]. Ninety-one patients were followed up at a minimum of 36 months. The average follow-up was 57.6 months (range 36–72).

### Statistical methods

Statistical analysis was analyzed by SPSS. The chi-square test was used to assess differences between categorical data. Data were described as mean ± standard deviation. The significance level was set at a *P* value of less than 0.05.

## Results

### Functional results

The Rowe scores in the arthroscopic Bankart repair group was higher than the other two groups with statistical significance (*P* < 0.05). Latarjet was lower in the poor Rowe level (*P* < 0.05). Compared to joint motion, external rotations on the affected side were 81.3 ± 3.1°, 79.8 ± 2.5°, and 78.5 ± 3.5° with no statistical significance (*P* > 0.05). No significant association was found between other outcome measures (Table 2).

**Table 2:** Functional results

Variable	Bankart (*n* = 53)	Latarjet (*n* = 52)	Capsular shift (*n* = 45)
ASES (mean ± SD)	92.12 ± 1.83	91.54 ± 2.38	92.41 ± 1.81
UCLA (mean ± SD)	29.40 ± 1.12	31.83 ± 1.35	31.13 ± 1.62
Hyperlaxity (ER > 85°) (%)	41 (77.4%)	38 (73.1%)	33 (73.3%)
Rowe score	92.36 ± 1.51	96.23 ± 2.10*	93.22 ± 2.31
Rowe level			
Excellent (90–100) (%)	27 (50.9%)	28 (53.8%)	24 (53.3%)
Good (75–89) (%)	18 (34.0%)	20 (38.5%)	15 (33.3%)
Fair (40–74) (%)	3 (5.7%)	2 (3.8%)	3 (6.7%)
Poor (0–39) (%)	5 (9.4%)	2 (3.8%)*	3 (6.7%)

*ASES* American Shoulder and Elbow Joint Surgery Association shoulder joint score, *ER* external rotation

*P < 0.05

### Subjective results

Interestingly, there were no significant differences in satisfaction among these groups (*P* > 0.05). However, the analysis showed better subjective results after Latarjet with respect to SSV Sport. The SSV Sport in the Latarjet group was superior to the Bankart group. By comparison, the capsular shift group had the lowest value (*P* < 0.05). Similar to the previous result, the capsular shift seemed to score the worst values in SSV (*P* < 0.05), as shown in Table 3.

**Table 3:** Subjective results

Variable	Bankart (*n* = 53)	Latarjet (*n* = 52)	Capsular shift (*n* = 45)
Very satisfied + satisfied (%)	47 (88.7%)	48 (92.3%)	37 (82.2%)
SSV (%)	50 (10–100)	50 (30–100)	39 (10–100)*
SSV Sport (%)	41 (0–100)*	44 (0–100)*	33 (0–100)*

*SSV* subjective shoulder value, *SSV Sport* subjective shoulder value for sport practice

*P < 0.05

### Complication

Five patients had a temporary postoperative complication. In the arthroscopic Bankart repair group, there were one postoperative hematoma along the arm and one transient musculocutaneous nerve palsy. In the open Latarjet group, there were two postoperative hematomas along the axillary fold and arm. In the capsular shift group, there was just one case who had a transient musculocutaneous nerve palsy. All postoperative hematomas were resorbed spontaneously after 6 weeks, and all cases who had a transient musculocutaneous nerve palsy recovered spontaneously after 6 months. Of course, none of them had any residual sequelae.

### Recurrence

One patient in the arthroscopic Bankart group (2.8%) and one in the capsular shift group (3.3%) had a postoperative recurrence. The former occurred after 2 years when playing basketball, and the latter was caused by a fall after 17 months. The Latarjet group had no recurrence.

## Discussion

With the development of arthroscopy, arthroscopic Bankart repair has already been widely used to treat recurrent anterior shoulder instability, which took away open surgery as the gold standard. To our knowledge, existing reports hold different attitudes, causing a fair amount of confusion, so that surgeon relied more on their experience due to the lack of guidelines.

Interestingly, our study found that patients undergoing Latarjet procedures could achieve significantly better stability, subjective perception for sport practice, and lower recurrence compared with the other two. This study also demonstrated that Bankart repair had no advantages in terms of external rotation and other outcome measure scores comparing open surgical procedures. In addition, the operation of Bankart repair did not decrease the number of complications from our study. However, considering that our study was still limited by sample size, we cannot tell whether differences of complications requiring reoperation remained statistically significant.

Several prior reports also revealed that open surgery appeared with better results, while opposite results were reported by others. In our study [11, 12], we used the Rowe score to assess postoperative stability, which contained stability, motion, and function. In terms of postoperative stability, Latarjet procedures may be more advisable [13]. We considered that the triple effect of anterior glenoid augmentation, the capsular repair, and the sling effect of the conjoint tendon may strengthen the efficacy, especially in significant structural bone deficits. But Latarjet procedures were not without defects. Matthes et al. have found the loss of elevation or internal and external rotation after the Latarjet procedure [14]. Facts proved that it coincided with our clinical practice despite a shortage of comparative researches. By contrast, Petrera et al. reported that patients undergoing modern arthroscopic Bankart repair showed better return to sport compared with open technique [15]. Uehara et al. also reported a similar result about Bankart repair and return to previous activity level [16].

Besides, it was also controversial that the loss of range of motion (ROM) after arthroscopic Bankart repair was minor to that after open capsular repair [17]. Some meta-analysis studies reported that Bankart repair can provide better recovery of ER at 90° abduction [18]. However, other studies indicated no significant difference in loss of ROM [19]. We compared the number of perioperative hyperlaxity (ER > 85°), and then, there was no difference in ER. However, we did not measure specific changes of angle, resulting in a certain error due to their uncertainty.

In the view of many surgeons, there was a lower risk of complications for Bankart repair compared with open surgery [20]. However, our study did not support this viewpoint. The incidence of complications of Bankart repair was higher than the others. Fortunately, there were two postoperative hematomas along the axillary fold and arm without loosening of fixation or small coracoid in the Latarjet group. Some reports revealed that the majority of complications of Latarjet procedure were related to a technical error and implant failure [7]. In such cases, some studies suggested choosing another surgical to avoid this complication due to lack of appropriate screwing fixation. According to our experience, a rigorous selection of patients who had small coracoid was the key to the success of Latarjet procedure [21]. For Bankart repair, the current large amount of researches argued that Bankart repair had fewer associated complications, including infection, nerve palsy, and internal rotation contractures [7]. This view was not supported in our study.

With respect to recurrence, our recurrence rate was 2.8% in the arthroscopic Bankart group, 0% in the Latarjet group, and 3.3% in the capsular shift group. We were delighted with this outcome in comparison with the classical arthroscopic Bankart surgery varying from 8 to 64% [7]. Of these patients, one undergoing Bankart repair had re-dislocation after 2 years when playing basketball and one undergoing capsular shift occurred at a fall. It indicated the potential factors of dislocation still existed after operation. For capsular shift, despite the good results from the beginning, long-term effect (maybe over 5 years) was still uncertain according to the paper by Hovelius et al. [22]. Burkhart and De Beer reported that glenoid bone loss greater than 25% led to a high risk of recurrence for arthroscopic Bankart repair, whereas recurrence was only 4% without bone loss [23]. Hence, we recommended Latarjet procedure to treat re-dislocation patients who had a higher request for activities and the absence of bone loss.

Overall, all three treatments proved to be effective in improving shoulder functional status and reducing symptoms. All of them had a satisfying result, and 85 cases returned to sports at the preinjury level at the final follow-up. The number of patients with a permanent incapacity for work was negligible in three groups. We can see the same results from the paper by Mohtadi et al. [24]. However, it was clear from this study that Bankart repair, Latarjet procedures, and capsular shift still had a lot of room to develop in treatment concept and technique of anterior shoulder instability. Take Latarjet technique as an example, it often presented an obvious challenge for junior doctors. To some extent, we can diminish these confounding factors by improving technique and training. Even so, there was still a certain rate of failure for an experienced surgeon because we cannot predict the evolution of the variable graft healing when undergoing Latarjet procedure [25]. One interesting thing we found was that most Chinese patients, especially older patients, were more willing to use complimentary care modalities or Bankart repair due to rapid recovery. Actually, according to years of work experience, capsular shift can be a good alternative if facing anteroinferior or multidirectional type of shoulder, but not including severe bone defect [17, 26]. As for the limitation of the thesis, further study was still demanded in this field.

By combining the clinical practice, some suggestions can be offered here to determine which patient is best suited to each surgical. Arthroscopic Bankart repair has the advantages of mini-invasion and rapid recovery. Capsular shift offers the advantage of stabilizing multidirectional type of shoulder. Latarjet can provide greater stability with low recurrence. At the same time, open Latarjet better suits young active patients, especially those with contact sports.

## Conclusions

The results revealed that all treatments were effective in improving shoulder functional status and reducing symptoms. However, open Latarjet procedure is more reliable in terms of shoulder stability and subjective perception than the others. In clinical practice, we still need to choose the optimal operative management on each specific matter, and here, we sum up some experience as reference: (1) Arthroscopic Bankart repair has the advantages of mini-invasion and rapid recovery. (2) Capsular shift has the advantages of stabilizing anteroinferior or multidirectional type of shoulder. (3) Latarjet was more effective in reducing recurrence with higher stability and has better subjective perception.

## Data Availability

The datasets used and analyzed during the current study are available from the corresponding author on reasonable request.

## Abbreviations

ASES: The American Shoulder and Elbow Joint Surgery Association shoulder joint score
ER: External rotation
ISIS: Instability severity index score
ROM: Range of motion
SSV Sport: Subjective shoulder value for sport practice
SSV: Subjective shoulder value
UCLA score: University of California at Los Angeles scoring system
